# Molecular characterization, antimicrobial resistance and clinico-bioinformatics approaches to address the problem of extended-spectrum β-lactamase-producing *Escherichia coli* in western Saudi Arabia

**DOI:** 10.1038/s41598-018-33093-8

**Published:** 2018-10-04

**Authors:** Muhammad Yasir, Abeer M. Ajlan, Shazi Shakil, Asif A. Jiman-Fatani, Saad B. Almasaudi, Muhammad Farman, Zainah M. Baazeem, Rnda Baabdullah, Maha Alawi, Nabeela Al-Abdullah, Nashat A. Ismaeel, Hani A. Shukri, Esam I. Azhar

**Affiliations:** 1 Special Infectious Agents Unit, King Fahd Medical Research Center, King Abdulaziz University, Jeddah, Saudi Arabia; 2 Biology Department, Faculty of Science, King Abdulaziz University, Jeddah, 21589 Saudi Arabia; 3 Center of Innovation in Personalized Medicine, King Abdulaziz University, Jeddah, Saudi Arabia; 4 Center of Excellence in Genomic Medicine Research, King Abdulaziz University, Jeddah, Saudi Arabia; 5 Department of Medical Laboratory Technology, Faculty of Applied Medical Sciences, King Abdulaziz University, Jeddah, Saudi Arabia; 6 Department of Medical Microbiology and Parasitology, Faculty of Medicine, King Abdulaziz University, Jeddah, Saudi Arabia; 7 Clinical and molecular microbiology laboratories King Abdulaziz University Hospital, Jeddah, Saudi Arabia; 8 Infection Control & Environmental Health Unit, King Abdulaziz University Hospital, King Abdulaziz University, Jeddah, Saudi Arabia; 9 Department of Public Health, Faculty of Nursing, King Abdulaziz University, Jeddah, Saudi Arabia

## Abstract

The goal of this study was to genotypically characterize extended-spectrum β-lactamase-producing *Escherichia coli* isolates from the western region of Saudi Arabia and to identify active antibiotics against these isolates using phenotypic and molecular modeling. In total, 211 ESBL-producing *E*. *coli* isolates recovered from heterogeneous clinical specimens were identified by MALDI-TOF. Thirty-two sequence types (STs) were identified from a multilocus sequence typing (MLST) analysis of ESBL-producing *E*. *coli*, including a novel ST (ST8162). The most common ST in the Saudi and expatriate population was ST131, followed by ST38. All the isolates were multidrug resistant (MDR), and >95% of the isolates were resistant to third-generation (ceftriaxone and ceftazidime) and fourth-generation (cefepime) cephalosporins. The ESBL-positive *E*. *coli* isolates primarily harbored the *bla*_CTX-M_ and *bla*_TEM_ genes. No resistance was observed against the carbapenem antibiotic group. All the ESBL-producing *E*. *coli* isolates were observed to be susceptible to a ceftazidime/avibactam combination. Molecular interaction analyses of the docked complexes revealed the amino acid residues crucial for the binding of antibiotics and inhibitors to the modeled CTX-M-15 enzyme. Importantly, avibactam displayed the most robust interaction with CTX-M-15 among the tested inhibitors in the docked state (∆G = −6.6 kcal/mol). The binding free energy values for clavulanate, tazobactam and sulbactam were determined to be −5.7, −5.9 and −5.2 kcal/mol, respectively. Overall, the study concludes that ‘ceftazidime along with avibactam’ should be carefully used as a treatment option against only carbapenem-resistant MDR ESBL-producing *E*. *coli* in this region.

## Introduction

Antimicrobial resistance is indiscriminate and is growing across different bacterial groups and against all antibiotic classes, impacting every region and country in the world. Particularly in the present infectious disease era, multidrug-resistant Gram-negative bacteria are a major therapeutic challenge in both hospital^1,2^ and community settings^3,4^. Out of 6 ESKAPE pathogens (recognized as particularly troubling), four are Gram-negative bacteria^5^. Thus, the attention of the scientific community has shifted to studying antibiotic-resistant Gram-negative bacteria^6^. Members of the family Enterobacteriaceae commonly produce extended-spectrum β-lactamases (ESBLs) that confer resistance to the advanced generation of cephalosporins and may lead to therapeutic dead ends. ESBLs are of great concern because they are often plasmid-associated. These plasmids often carry antimicrobial resistance genes for other antibiotics, such as fluoroquinolones, aminoglycosides, tetracyclines, sulfamethoxazole-trimethoprim and chloramphenicol. Importantly, there is a potential risk of cross-species dissemination of these plasmids, resulting in multiple antibiotic resistance genes being spread to different bacterial species^7^. Infections from extended-spectrum β-lactamase-producing *E*. *coli* are often preceded by asymptomatic carriage^8^. Moreover, hospital acquired infections due to ESBL-producing *E*. *coli* are common, and several nosocomial outbreaks have been reported in different geographical regions^9^. Recently, several studies from Saudi Arabia have reported the ESBL-producing Enterobacteriaceae *Klebsiella pneumoniae* and *E*. *coli*, primarily from Riyadh^10,11^. However, very limited data are available regarding the genetic background and clonal type of invasive *E*. *coli* from this region.

With respect to ESBLs, *bla*_CTX-M_ have become the most commonly detected ESBL genotype in different geographical locations worldwide^12^. In a recent study, Bindayna *et al*. identified *bla*_CTX-M_ as the predominant genotype among ESBL-producing bacteria in Saudi Arabia^13^. The change in the enzymatic activities of *bla*_CTX-M_ that have led to the evolution of additional variants may be due to point mutations within or outside the active site omega loop, i.e., amino acid positions 161 to 179^14^. Identification of the amino acid residues that are crucial to the interaction between *bla*_CTX-M_ variants (the enzymes produced by resistant bacteria) and target drugs (the drug hydrolyzed by these bacterial enzymes) is a problem of deep scientific interest. Information on this subject would be useful for the development of *bla*_CTX-M_-resistant antibiotics in the future. Variants of this bacterial enzyme are emerging at a fast pace. Up to 172 *bla*_CTX-M_ variants have already been reported (http://www.lahey.org/studies/other.asp#table1; accessed on 27/03/2018). Thus, there is an urgent need to design a versatile *bla*_CTX-M_ inhibitor that can inhibit most of the emerging variants of this enzyme, if not all. Moreover, continuous surveillance is needed because the evolution of newer *bla*_CTX-M_ enzyme variants with altered and expanded substrate (antibiotic) profiles has now become an everyday phenomenon^15^.

In this study, ESBL-producing *E*. *coli* isolates collected from one of the major territory hospitals of the King Abdulaziz University, which serves Jeddah and the peripheral area, were screened for antimicrobial susceptibility and *bla*_CTX-M_ genotypes. A multilocus sequence typing (MLST) analysis was performed to identify the common sequence types (STs) of ESBL-producing *E*. *coli* circulating in this region, as approximately half of the population are expatriates from developing countries in addition to natives of Jeddah, which is the primary entry point for 30 million pilgrims every year from all over the world. In addition, modeling studies were performed to allow the best therapy against *bla*_CTX-M_-producing bacteria to be suggested to local health care facilities.

## Results

### Demographic and Clinical Data Analysis

A total of 211 nonduplicate ESBL-producing *E*. *coli* clinical isolates that were collected during 2014–2015 and identified by MALDI-TOF were characterized in this study. The isolates were obtained at a relatively highly percentage from inpatients (75.4%) followed by outpatients (24.6%). No significant difference (p > 0.05) was observed in the gender of patients, with 115 (54.5%) and 96 (45.5%) being female and male, respectively (Table 1). The average age of the patients was 48 years, ranging from 0.1–96 years with a median age of 55 years. A relatively higher percentage of isolates was obtained from patients older than 50 years of age (54.9%), among whom 85 were inpatients (55.6%). Importantly, 10% of the ESBL-producing *E*. *coil* isolates were from neonates <1 year of age (Table 1). The strains were primarily isolated from Saudi patients (42.7%), as well as Yemeni (22.7%), Sudanese (6.2%), Palestinian (4.7%) and Pakistani (4.7%) expatriates, with the remaining 13.3% isolates recovered from patients of 13 other nationalities. The strains were cultured from heterogeneous clinical specimens, mostly from urine (36.5%) and urine catheters (27.9%), followed by wound swabs (10.4%) and blood samples (9%) (Table 1). The results of a chi-square test revealed an association between patient and specimen types (p = 0.001).

**Table 1:** Distribution of ESBL-positive *E*. *coli* isolates according to demographic and clinical data.

	Source	Total No.	Inp n = 159 (75.4%)	Outp n = 52 (24.6%)	p-value	HA n = 88 (41.7%)	CA n = 123 (58.3%)	p-value
Gender	Male	96	83 (52.2)	13 (25.0)	0.001	46 (52.3)	50 (40.7)	0.095
	Female	115	76 (47.8)	39 (75.0)		42 (47.7)	73 (59.3)	
Age	≤1	21	20 (12.6)	1 (1.9)	0.015	11 (12.5)	10 (8.6)	0.438
	2–11	14	11 (6.9)	3 (5.8)		5 (5.7)	9 (7.3)	
	12–18	2	1 (0.6)	1 (1.9)		0	2 (1.6)	
	19–50	58	36 (22.6)	22 (42.3)		22 (25.0)	36 (29.3)	
	>50	116	91 (57.2)	25 (48.1)		50 (56.8)	66 (53.7)	
Nationality	Saudi	90	65 (40.9)	25 (48.1)	0.057	40 (45.5)	50 (40.7)	0.227
	Yemeni	48	34 (21.4)	14 (26.9)		17 (19.3)	31 (25.2)	
	Sudanese	13	13 (8.2)	0		7 (8.0)	6 (4.9)	
	Palestinian	10	7 (4.4)	3 (5.8)		2 (2.3)	8 (6.5)	
	Pakistani	10	9 (5.7)	1 (1.9)		6 (6.8)	4 (3.3)	
	Ethiopian	6	4 (2.5)	2 (3.8)		3 (3.4)	3 (2.4)	
	Somalian	6	6 (3.8)	0		3 (3.4)	3 (2.4)	
	Others	28	21 (13.2)	7 (13.5)		17 (19.3)	24 (19.5)	
Specimens Type	Urine-midstream	77	40 (25.1)	37 (71.2)	0.001	27 (30.7)	50 (40.7)	0.034
	Urine-catheter	59	50 (31.4)	9 (17.3)		19 (21.6)	40 (32.5)	
	Wound swab	22	20 (12.6)	2 (3.8)		14 (15.9)	8 (6.5)	
	Blood	19	18 (11.3)	1 (1.9)		9 (10.2)	10 (8.1)	
	Others	34	31 (19.5)	3 (5.8)		29 (33.0)	15 (12.2)	

Inp, Inpatient; Outp, Outpatient; HA, Hospital acquired; CA, Community acquired.

The majority of the ESBL-positive *E*. *coli* infections were of community-onset (58.3%), and 41.7% were likely nosocomial infections acquired by the patients during three-day admissions in different hospital units; such infections were primarily identified from patients in male and female medical and surgical wards and pediatric wards. Among the patients with nosocomial infections, 25.6% were cancer patients. ESBL-positive *E*. *coli* isolates from nosocomial infections were primarily identified from midstream urine samples (30.7%), urine catheters (21.6%) and wound swabs (15.9%) (Table 1). The community-onset patients were primarily outpatients (42.3%) from the emergency room (26.8%). In more than 85% of patients, an ESBL-positive *E*. *coli* infection was not the primary diseases. Among these patients, ESBL positive *E*. *coli* were primarily isolated from the malignancy, sepsis, urinary tract infection (UTI) and other renal diseases of patients (Supplementary Fig. S1).

### Antimicrobial Susceptibility Analysis

**Figure 1:**
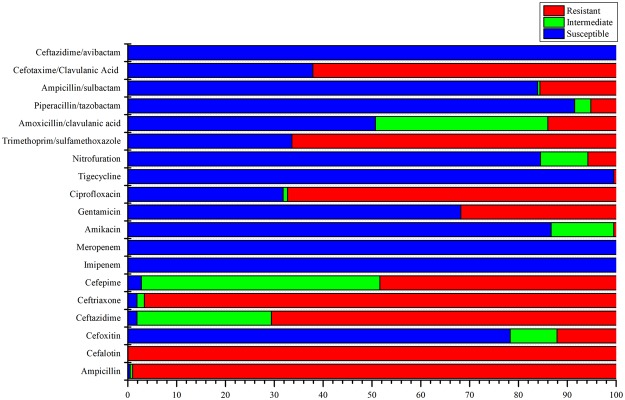
Antimicrobial resistance and sensitivity profile of the tested antibiotics among the studied ESBL-positive *E*. *coli* isolates. The x-axis values are expressed in percentage.

All of the tested *E*. *coli* isolates were determined to be multidrug resistant (MDR), and >60% isolates were revealed as extensively drug-resistant (XDR). None of the isolates were recorded as pan-drug resistant (PDR) in this study. In total, 97.6% of isolates were resistant to ≥5 of the tested antibiotics. Among them, 16 isolates were resistant to ≥10 antibiotics. The isolates Ec51 and Ec128, which were recovered from urine catheter and blood samples from Pakistani and Sudanese sepsis patients, respectively, were resistant to the 12 tested antibiotics. Ampicillin resistance was detected in the 99.5% of isolates, with observed MICs of ≥32 µg/ml (Fig. 1). All the tested isolates were resistant to the first-generation cephalosporin cefalotin (8 to ≥64 µg/ml), while >95% of isolates were resistant or intermediately resistant to ceftriaxone, ceftazidime and cefepime. Only 21.3% isolates were resistant or intermediately resistant to the second-generation cephalosporin cefoxitin, with observed MICs of 16–64 µg/ml. All the isolates were susceptible to carbapenem antibiotics (imipenem and meropenem). Other than β-lactam antibiotics, the highest resistance was observed against ciprofloxacin (68.2%) and trimethoprim/sulfamethoxazole (66.4%), with observed MICs of ≥4 and ≥320 µg/ml, respectively (Fig. 1). The lowest antibiotic resistance was observed against amikacin and nitrofurantoin. One ESBL-positive *E*. *coli* isolate was resistant to tigecycline, with an MIC of ≥8 µg/ml. No specific association was observed among the disease types and resistances to particular antibiotics in the ESBL-producing *E*. *coli* isolates. However, resistance to trimethoprim/sulfamethoxazole was increased to 79% in the cancer patients. In total, 70 different patterns of antibiotic resistance were observed in the 211 isolates, which included 30 groups and 40 unique patterns. The dominant antibiotype pattern (P1) was observed for 26 ESBL-positive *E*. *coli* isolates that exhibited resistance to 7 antibiotics and sensitivity to 9 antibiotics (Supplementary Fig. S2). The second antibiotype pattern (P2) was identified in 17 isolates that were resistant to 5 antibiotics and susceptible to 11 antibiotics. The third antibiotype pattern (P3) was identified in 15 isolates that were resistant to 9 antibiotics (Supplementary Fig. S2). The dominant antibiotypes were primarily identified in the inpatients from the >50 years old age group. The other antibiotype patterns were observed in ≤10 isolates. Most importantly, all the ESBL-producing *E*. *coli* isolates were determined to be susceptible to the ceftazidime/avibactam combination (Fig. 1).

### β-lactamase Resistance Genes Genotyping

The ESBL-positive *E*. *coli* isolates primarily harbored the *bla*_CTX-M_ (95.3%) and *bla*_TEM_ (83.9%) genes. The *bla*_OXA_ and *bla*_SHV_ genes were only detected in 6.6 and 5.2% isolates, respectively (Supplementary Fig. S3). In total, 82.5% isolates carried more than one β-lactamase gene. The combination of *bla*_CTX-M_ and *bla*_TEM_ (79.1%) was frequently observed in the ESBL-positive *E*. *coli* isolates. The combination of *bla*_CTX-M_, *bla*_TEM_ and *bla*_SHV_ was observed in only 2.4% of isolates. The *bla*_CTX-M_ isolates primarily belonged to the group *bla*_CTX-M-1_ (74.6%), followed by *bla*_CTX-M-9_ (20.4%). The groups *bla*_CTX-M-8/25_ and *bla*_CTX-M-2_ were detected only in 2.5 and 1.5% of isolates respectively.

### MLST Analysis and Identification of Clonal Complex Groups

**Figure 2:**
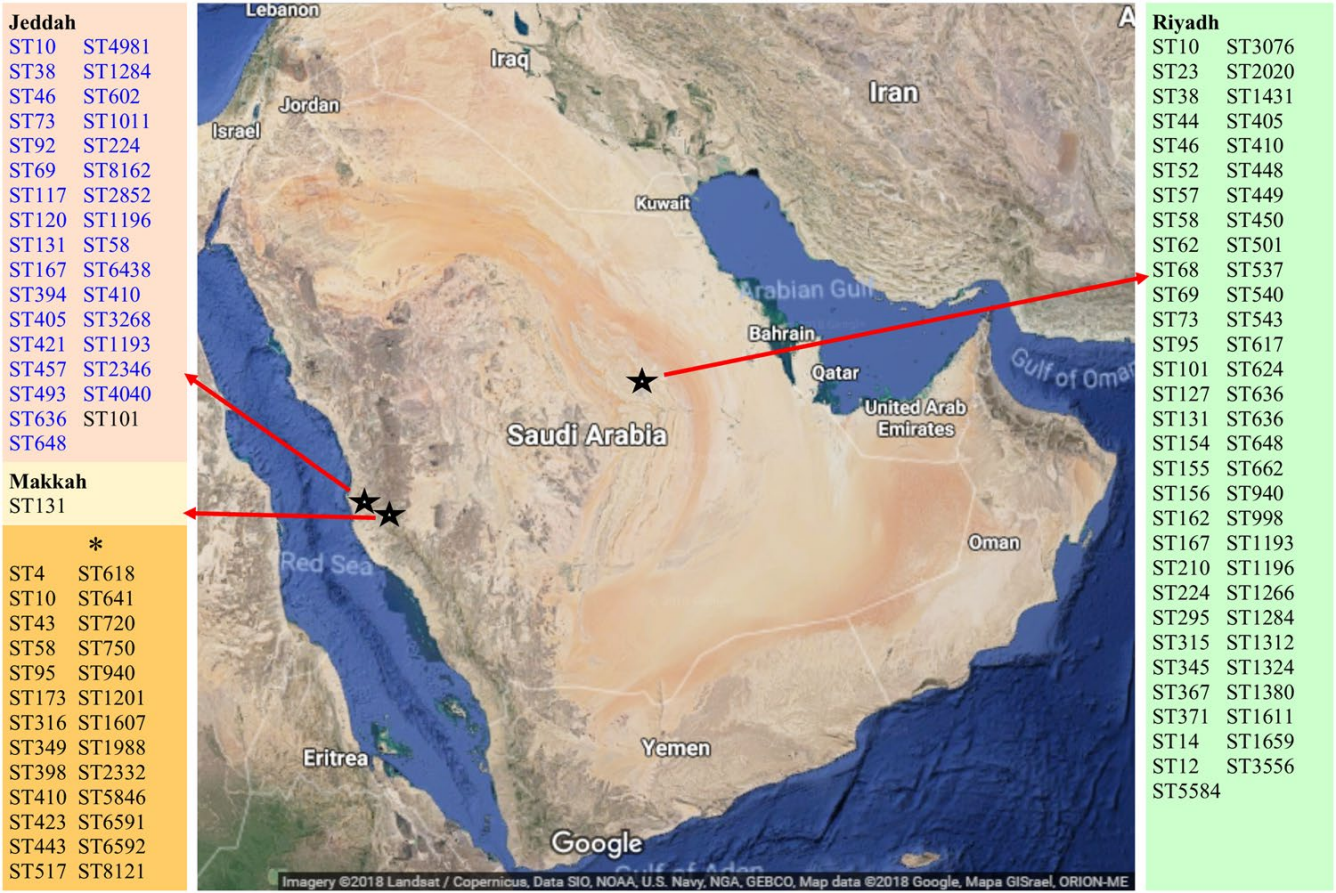
Regional distribution of *E*. *coli* STs detected in Saudi Arabia. The blue labeled STs from Jeddah were detected in this study. *Indicates the STs were previously recovered from food samples and their location in Saudi Arabia was not reported. The map in the figure was obtained from Map data@2018 Google.

**Figure 3:**
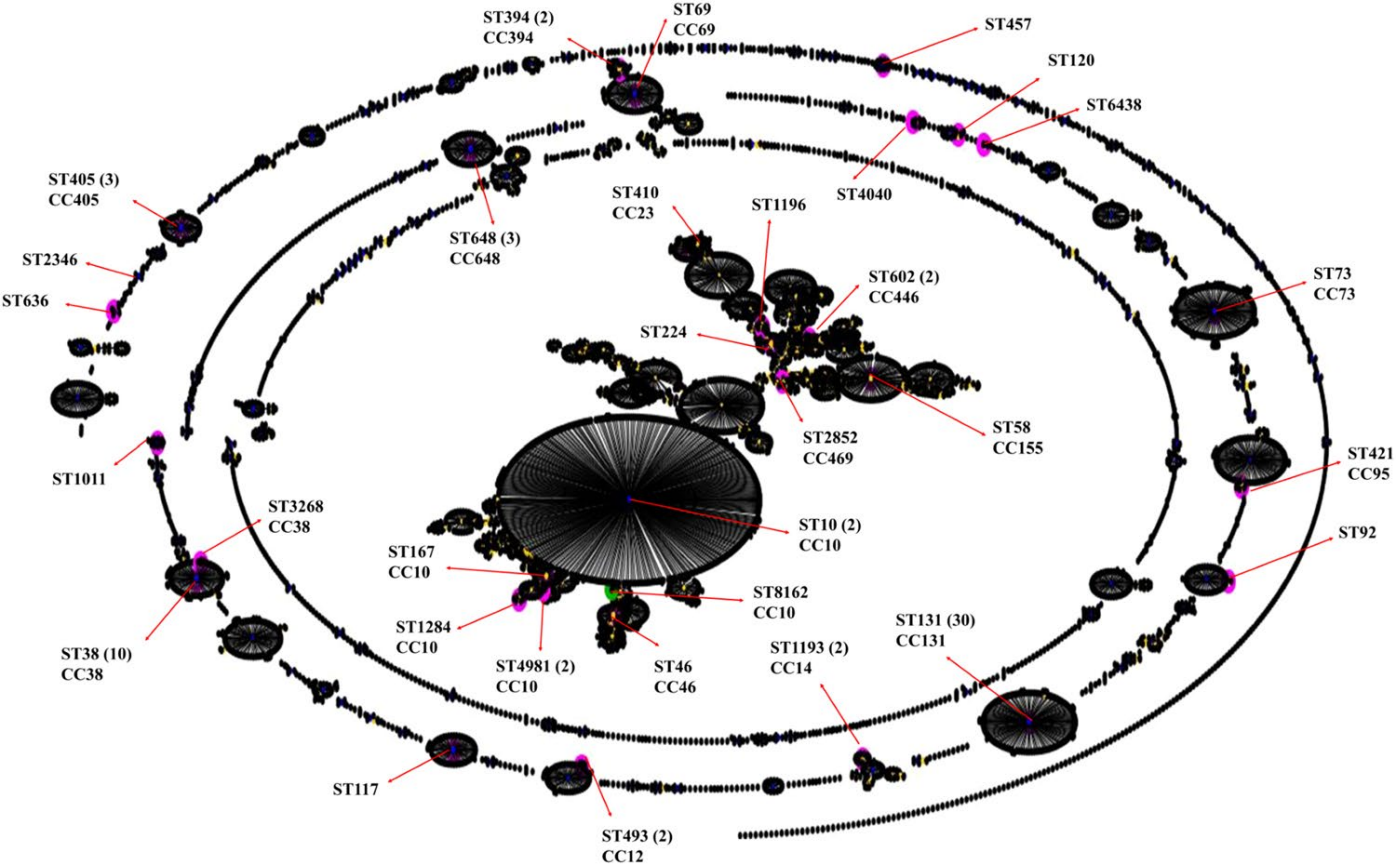
eBURST analysis of multilocus sequence typed ESBL-positive *E*. *coli* isolates. The pink nodes indicate the STs detected in this study that were present in the MLST database. The green node indicates the novel ST detected in this study.

ERIC-PCR produced varying amplification products according to molecular weights observed by gel electrophoresis. The amplicons were between 150 and 7,000 bp, with multiple bands (between 2 to 12 bands) observed for each isolate (Supplementary Fig. S4). The isolates were divided to 80 clusters at distance of 0.5, and a representative isolate from each clade was processed for MLST. Overall, 32 unique STs were detected among the 80 isolates using the allelic profile of the seven housekeeping genes, including a novel ST8162 (Fig. 2). In addition, 16 *E*. *coli* STs were identified in this study that were not previously reported from Saudi Arabia. The predominant ST identified was ST131 (37.5%, n = 30), followed by ST38 (12.5%, n = 10). Other identified STs were detected in ≤3 of ESBL-positive *E*. *coli* isolates, while 22 identified STs were comprised of a single isolate. eBURST resolve the 32 unique allelic profiles of the isolates into 3 groups and 26 singletons with a single locus variant and 4 groups and 20 singletons with a double locus variant (Fig. 3). In contrast, the BLAST analysis with the EnteroBase database grouped the 32 STs into 14 clonal complexes (CC), including the major CCs CC131 and CC38. The allelic profiles of 6 STs linked with 4 different CCs with a single locus variant, such as ST4981, ST1284 and ST8162, which were linked with CC10 with a single locus variant. The allelic profile of the 11 STs were identified as singletons.

**Figure 4:**
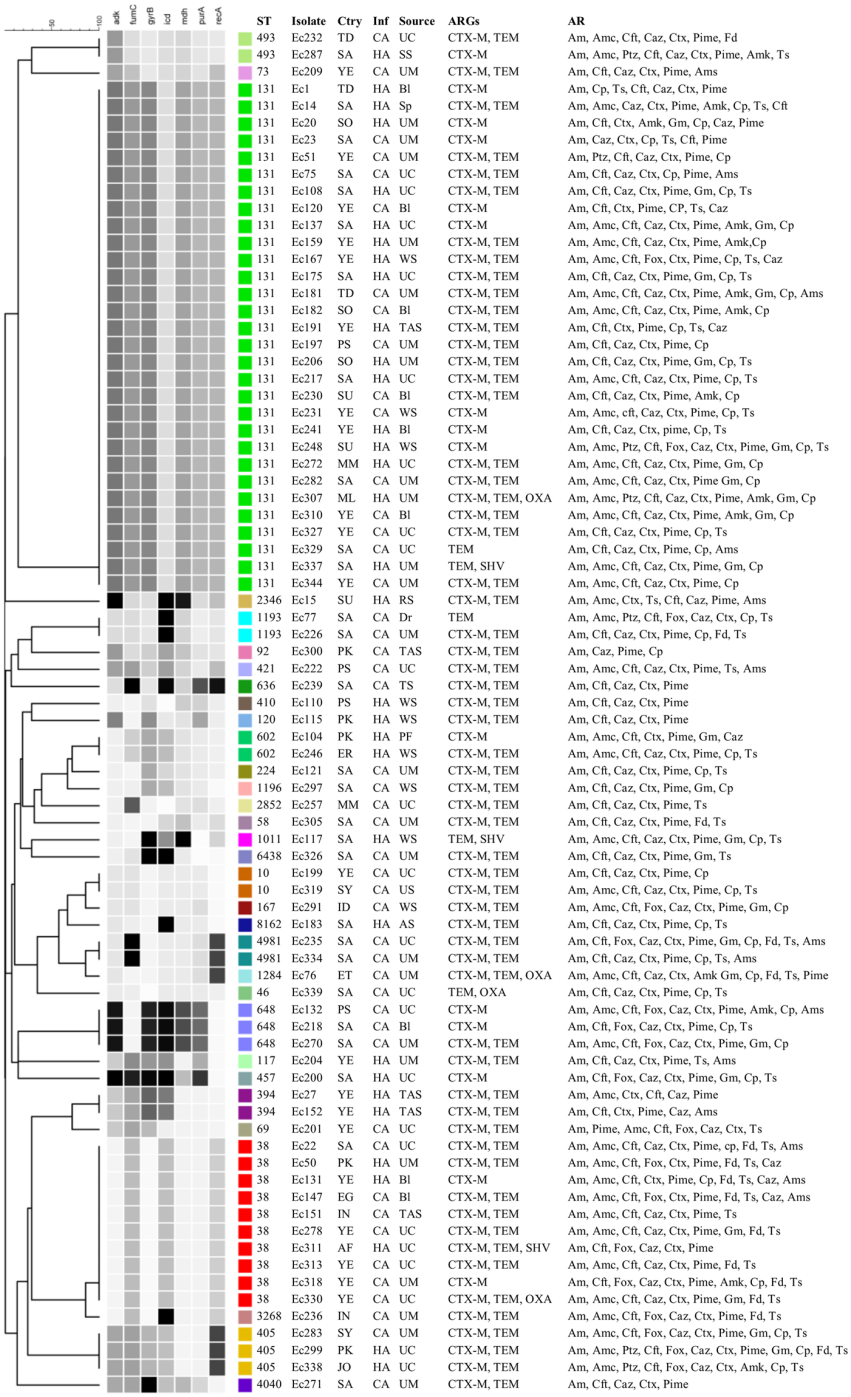
UPGMA dendrogram from the pattern of pairwise differences in alleles that revealed the genetic relationships of STs among the ESBL-positive *E*. *coli* isolates, along with the country (Ctry), infection (Inf), specimen source, antimicrobial resistance genes (ARGs) and antibiotic resistances (AR) information. HA, hospital acquired; CA, community acquired; UC, urine-catheter; UM, urine-midstream; SS, skin swab; Bl, blood; Dr, drain; Sp, sputum; WS, wound swab; TAS, tracheal aspirate; TS, tissue swab; RS, rectal swab; AS, ascitic fluid; PF, peritoneal fluid.

The predominantly identified CC131 and CCT38 were significantly recovered from the inpatients. However, no significant association was observed for these CCs with either community acquired or hospital acquired infections or with disease type (Fig. 4). The CC131 isolates were primarily cultured from midstream urine samples or urine catheters followed by blood specimens (Fig. 4). The two CC394 isolates were specifically recovered from tracheal aspirates of intensive care patients who acquired nosocomial infections of ESBL-positive *E*. *coli*, whereas the isolates associated with CC10 were associated with the community acquired infections. CC648 was observed in the CA cases and CC405 was observed in both HA and CA cases. The same STs, such as ST131, ST394, ST405 and ST648, were recovered from multiple hospital wards and intensive care units and patient with different diseases. No specific association was observed between clinically significant CCs and clinical specimens. In total, 14 different STs, primarily ST131 (44%), were identified from the midstream urine samples. In the urine catheter specimens, 12 different STs were identified, including the 5 STs detected in the midstream urine samples. In addition, 3 different STs were identified in blood specimens, and ST131 was primarily identified in blood. No specific association was observed for the other clinically significant CCs and clinical specimens. The 32 STs were identified from patients of 17 different nationalities, and the same ST was observed in patients of different nationalities, suggesting the diversity and cotransfer of the STs among patients from different nationalities in the health care system of Saudi Arabia. The new ST (ST8162) was recovered from a Saudi patient.

All the isolates associated with CC131 and CC10 were resistant to ampicillin, cefalotin, ceftazidime, ceftriaxone, cefepime and ciprofloxacin and were sensitive to imipenem, meropenem and tigecycline (Fig. 4). In addition, all the CC131 isolates were sensitive to nitrofurantoin. In contrast to CC131 and CC10, the majority of isolates associated with CC38 were sensitive to ciprofloxacin and resistant to nitrofurantoin and trimethoprim/sulfamethoxazole. The *bla*_CTX-M_ gene was present in 93.3% of the ST131 isolates, and this group primarily harbored *bla*_CTX-M-1_ (60.3%), whereas 66.6% of ST131 isolates contained both the *bla*_CTX-M_ and *bla*_TEM_ genes (Fig. 4). None of the ST131 isolates contained the *bla*_CTX-M-8/2_ and *bla*_CTX-M-2_ group genes. All the isolates associated with CC38 carried *bla*_CTX-M_ genes, primarily *bla*_CTXM-1_, followed by *bla*_CTX-M-9_ genes. All the isolates associated with CC10 carried both *bla*_CTX-M_ and *bla*_TEM_ genes.

### Molecular Modeling and Docking

**Figure 5:**
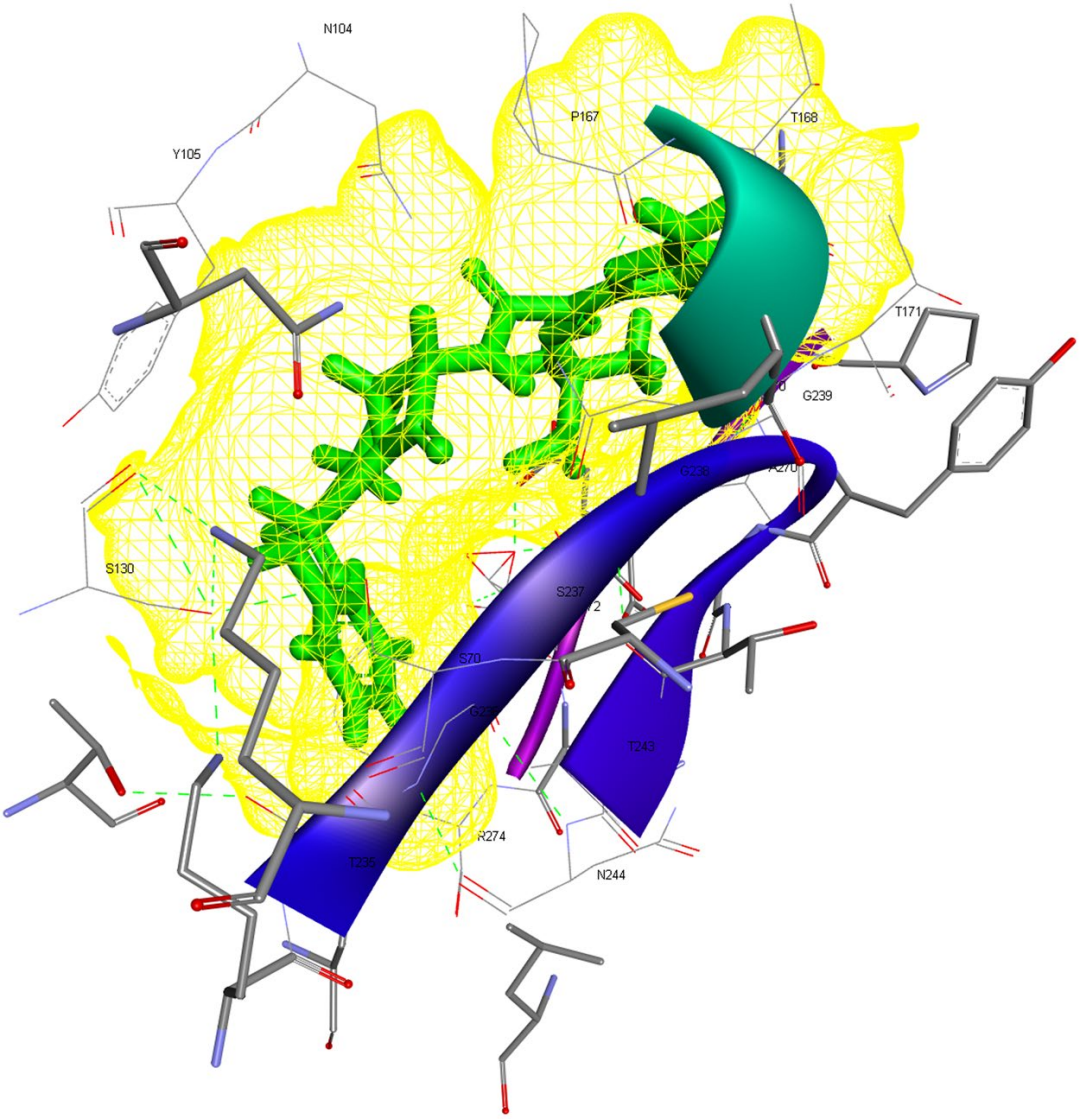
Wire-mesh representation of the ceftazidime-CTX-M-15 docked complex. Interacting amino acid residues of the binding pocket are labeled.

**Figure 6:**
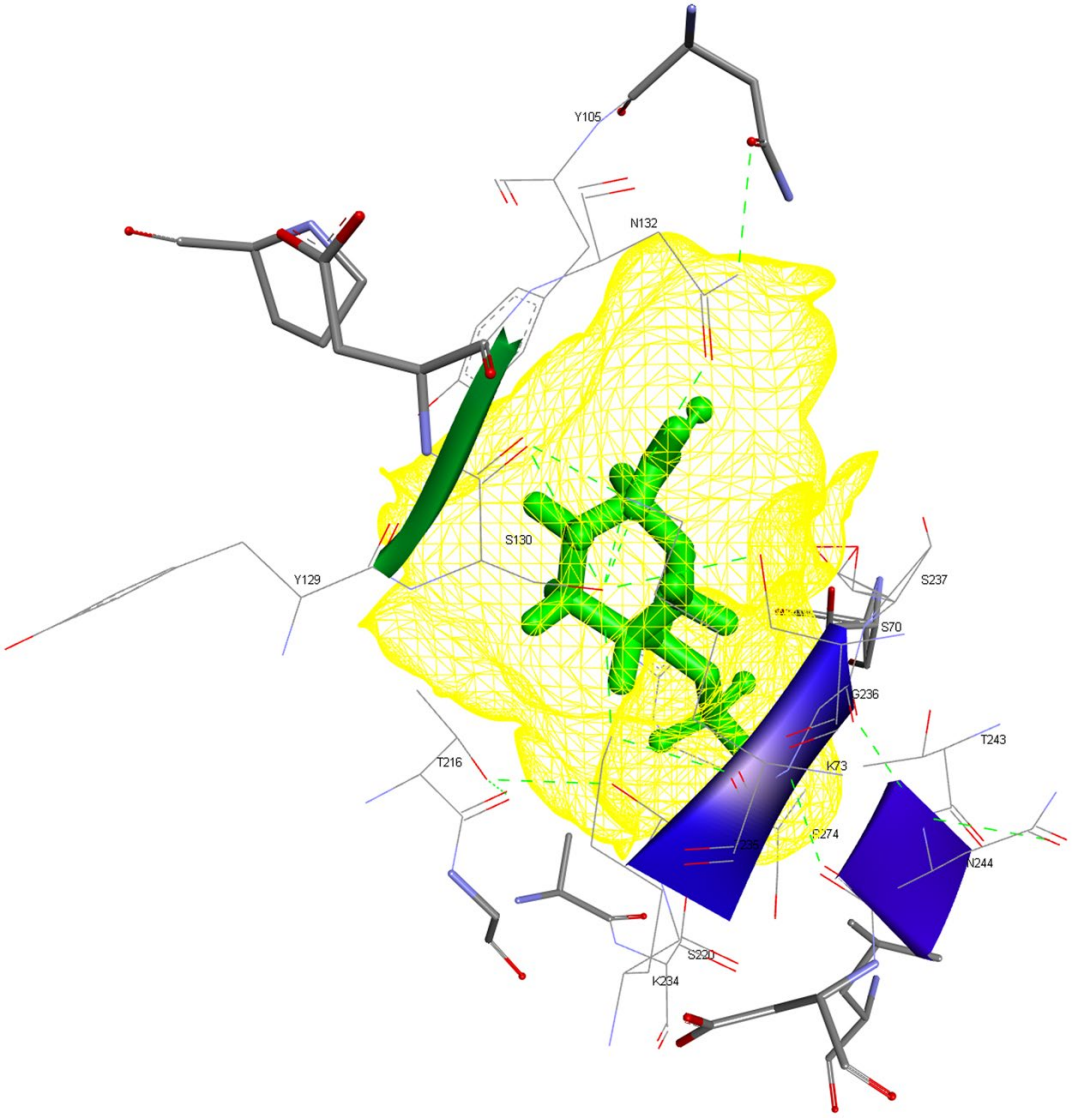
Wire-mesh representation of the avibactam-CTX-M-15 docked complex. Interacting amino acid residues of the binding pocket are labeled.

The results of the BLAST analysis revealed that CTX-M-15 was the most prevalent variant in our study. In addition, a multiple sequence alignment by CLUSTALW indicated that within the CTX-M-15 variants, there was no noteworthy difference in the amino acid residues, particularly in those residues which constituted the “active site omega loop”. Thus, we randomly picked a CTX-M-15 sequence for molecular modeling, with the bacteria harboring this sequence having displayed significant resistance to advanced generation cefalosporins in the MIC and susceptibility tests. Briefly, the results of model validation programs indicated the CTX-M-15 model to be of ‘Good quality’ (Supplementary Figs S5 and S6). Molecular interaction analyses of the docked complexes revealed the amino acid residues crucial to the binding of antibiotics and inhibitors to the modeled CTX-M-15. Figure 5 represents the docking pose and the interacting amino acid residues for the CTX-M-15-ceftazidime-complex (Fig. 5). In contrast, avibactam displayed the most robust interaction with CTX-M-15 among the inhibitors in the docked state (ΔG = −6.6 kcal/mol). The corresponding pose and interacting amino acid residues are shown in Fig. 6. The binding free energy values for clavulanate, tazobactam and sulbactam were determined to be −5.7, −5.9 and −5.2 kcal/mol, respectively.

## Discussion

In this study, we provide comprehensive antimicrobial resistance and molecular epidemiological profiles of ESBL-producing *E*. *coli* from the Jeddah region of Saudi Arabia. Previous studies from this region have investigated selected antibiotics and clonal groups, limiting their capacity to establish a broad profile. In this study, the majority of isolates were resistant to ampicillin and cephalosporin antibiotics (except cefoxitin), followed by fluoroquinolone and trimethoprim/sulfamethoxazole, which may be associated with self-medication and the common use of β-lactam antibiotics by humans in this region, and this is a trend in the surrounding countries and in the countries of origin of the expatriates. However, we did not detect any carbapenem resistant ESBL-producing *E*. *coli*, and low resistance was observed against amikacin, which could be used to combat *E*. *coli* infections in this region. Other studies from Saudi Arabia observed comparable levels of resistance in ESBL-producing *E*. *coli* recovered from UTI patients and other clinical specimens^11,16^. However, Al-Agamy recently reported the isolation of carbapenem-resistant *E*. *coli* from Riyadh hospitals, including *bla*_NDM-1_- and *bla*_OXA-48_-positive isolates^17^. The findings of this study show for the first time that the primary incidence of ESBL-producing *E*. *coli* infection in this area are community-onset infections.

Importantly, a marked diversity of clones was observed in the *E*. *coli* isolates from Saudi Arabia, and 32 distinct STs from 80 isolates were processed for MLST in this study. Consistent with worldwide reports, ST131 was the predominant ST identified, followed by ST38 and the isolates associated with CC10^11,18^. In the United States, 53% of ESBL-producing *E*. *coli* are associated with ST131^19^. Similarly, in the United Kingdom and France, 64 and 25% of community-acquired infections from *E*. *coli* belonged to ST131, respectively^20,21^. In previous studies, apart from ST131, STs, ST69, ST73 and ST95 were predominantly identified in a large collection of *E*. *coli* and accounted for 40–60% of UTI isolates^22,23^. In contrast to the previous local reports and studies from the western world, ST38 was the second dominant ST detected in this study. These results, together with those of published studies from Saudi Arabia, suggest the existence of variation in the range of ST131 in the eastern and western regions of Saudi Arabia and indicate a high percentage of ST131 in the Makkah region (≥30%) and in Riyadh (17.3%)^10,11^. Similarly, variation was observed in the relative percentage distribution of other commonly identified STs in the studies from the eastern and western regions of Saudi Arabia, such as ST73, which was detected at a relatively higher abundance in the eastern region of Saudi Arabia^10^. Prior to this study, 16 STs, including ST1284, ST92, ST120 and ST46, had not been previously reported from Saudi Arabia and were previously reported from pathogenic strains associated with different diseases worldwide. In addition, we identified a novel ST (ST8162) from a Saudi patient. However, caution should taken in applying the current finding of *E*. *coli* clonal distribution to the different geographical regions of Saudi Arabia, as the analysis is based on a limited number of available data. Thus, additional studies are required to understand the distribution of *E*. *coli* sequence types in Saudi Arabia. The high diversity in the *E*. *coli* clones may have arisen because approximately half of the population of Saudi Arabia are expatriates from developing countries, including Pakistan, India, Bangladesh, the Philippines and African countries where self-medication in patients is evident^24^.

The antibiotic resistance observed in this study with respect to the different STs were general patterns, with multidrug resistance particularly observed against ampicillin, cephalosporins, ciprofloxacin and trimethoprim/sulfamethoxazole, and the majority of isolates were sensitive to carbapenems. All the ST131-associated isolates were resistant to ciprofloxacin and were sensitive to imipenem, tigecycline and nitrofurantoin. In contrast, low resistance to ciprofloxacin and high resistance to nitrofurantoin was observed in the ST38 isolates. A disproportionate susceptibility to ciprofloxacin in ST131 isolates has been observed in different studies in various geographical regions. In North America, a 23–28% resistance to fluoroquinolone was reported in ST131 isolates, and background resistance was 27% in *E*. *coli*^25^. Similarly, a recent study from Australia observed a 21% fluoroquinolone resistance rate in ST131 isolates from a selected patient population with UTIs, with an estimated 41% of fluoroquinolone-resistant *E*. *coli* within the studied population^26,27^. Overall, the background ciprofloxacin resistance is high in our region compared to other countries where low rates of fluoroquinolone resistance in *E*. *coli* have been achieved through regulatory control of fluoroquinolone use in humans and animals^28^. We observed that the ST405, ST1284 and ST648 isolates were resistant to ≥nine antibiotics. However, the association between ST and the prospective resistance reasonably suggests that rapid identification of *E*. *coli* sequence types by molecular techniques might allow treatment to be optimized before the conventional antibiogram could be completed. This would be particularly important in the case of the commonly resistant ST131, as it would allow treatment to be switched, e.g., to a carbapenem in life-threatening infections, or to nitrofurantoin in uncomplicated UTI cases in Saudi Arabia.

The results of this study suggest that the ESBL genes, primarily *bla*_CTX-M_ and *bla*_TEM_, are widely scattered into the 32 STs, which has been confirmed by other reports^10,11^. The ST131 and ST38 isolates were mainly comprised of the *bla*_CTX-M_ group *bla*_CTX-M-1_ genes, followed by *bla*_CTC-M-9_. Recent studies have revealed a substantial increase in the number of *bla*_CTX-M_-producing *E*. *coli*, which is probably due to the central mechanisms of clonal expansion of *E*. *coli* ST131^29^. We have analyzed all ESBL-positive isolates for *bla*_CTX-M_ genes by a PCR assay. The results showed that almost all the isolates (99.5%) carried *bla*_CTX-M_. Our results, together with those of previous studies, indicates that the *bla*_CTX-M_ type is the most common ESBL resistance gene in Saudi Arabia; the rate is higher than that reported from Europe and is consistent with that of China^10,20,30^. At the same time, the primary *bla*_CTX-M_ group was *bla*_CTX-M-1_, which was confirmed by several other local studies^10^. In China, the primary *bla*_CTX-M-14_ and *bla*_CTX-M-15_ gene types were reported from *bla*_CTX-M_, and differences were observed in the distribution of *bla*_CTX-M-1_ and *bla*_CTX-M-9_, highlighting the existence of regional epidemiological features. The assay results for the *bla*_TEM_, *bla*_SHV_ and *bla*_OXA_ genes showed that *bla*_TEM_-positive *E coli* strains were also very common in the ESBL-producing *E*. *coli*, while the others were relatively rare, which is consistent with the results from previous studies conducted in China^31^. In addition, the positive rates of the *bla*_TEM_, *bla*_SHV_ and *bla*_OXA_ genes exhibited some differences among areas, indicating that there are some regional epidemiological characteristics. As the different ESBL resistance genes encode β-lactams with different hydrolysis capabilities, the different ESBL distributions should be considered with respect to antibacterial use.

Studies at the interface of clinical microbiology and bioinformatics are increasingly being used to address the problem of multiple antibiotic resistance in bacteria. The ensuing battle, i.e., antibiotics versus bacteria, has been acknowledged by the scientific community^32,33^. Consequently, we suggest that a combination of “ceftazidime and avibactam” could be the best therapy to tackle infections caused by carbapenems resistant Gram-negative bacteria among Saudi patients. This suggestion is supported by the findings of Flamm *et al*.^34^, where the authors reported that all of the screened ESBL-positive and meropenem-nonsusceptible *E*. *coli* and *K*. *pneumoniae* isolates displayed a ceftazidime-avibactam MIC of ≤4 μg/mL^34^. Furthermore, Castanheira *et al*.^35^ tested the activity of a ceftazidime-avibactam combination against Enterobacteriaceae from US hospitals and observed that it displayed commendable activity against the studied strains, including multiresistant KPC-producers^35^. We therefore implore and encourage the scientific community to recommend the judicious use of antibiotics to help restore bacterial susceptibility to agents such as bactrim and fluoroquinolones. If need be, the first-line agent for ESBL-producing *E*. *coli* should be a carbapenem such as ertapenem. It is suggested that ceftazidime-avibactam should be reserved for cases of Carbapenem-Resistant Enterobacteriaceae (CRE). Even in this situation, caution must be exercised, as there are reports of the emergence of ceftazidime-avibactam resistance among CRE with the increasing use of this novel agent^36^.

## Conclusion

The threat of multidrug resistant ESBL-producing *E*. *coli* is steadily increasing in Saudi Arabia. A markedly diverse clonal composition of the ESBL-producing *E*. *coli* isolates was observed in the western region of Saudi Arabia. A potential factor contributing to this observation may be the large expatriate population working in the metropolitan city of Jeddah and its surroundings. ST38 was identified as an emerging clone following ST131, and *bla*_CTX-M_ was identified as the primary genotype. The results obtained by microbiological and computational approaches reinforce the conclusion of the present study. Overall, the study concludes that ‘ceftazidime along with avibactam’ and carbapenems could be two promising treatment options against MDR ESBL-producing *E*. *coli* in this region. Carbapenems may be used as a first-line option against *E*. *coli* isolates resistant to cephalosporins, ciprofloxacin and gentamycin from this region. Furthermore, the results described herein may be useful to update the empirical antibiotic regimens in the health care facilities of Saudi Arabia. An effective antibiotic stewardship program and regular antibiotic resistance surveillance studies are required to avoid complications in the future management of clinical isolates of *E*. *coli-*producing ESBLs.

## Materials and Methods

### Sample collection

This study was conducted at the special infectious agent unit (SIAU) of King Fahd Medical Research Center (KFMRC) with the collaboration of the infection control unit and the clinical and molecular microbiology laboratory of King Abdulaziz University hospital (KAUH) in Jeddah. KAUH is an 845-bed teaching hospital of the King Abdulaziz University that primarily serves the western region of Saudi Arabia. This study was approved by the ethical research committee of the Faculty of Medicine at King Abdulaziz University under the reference number (148–14). The participants provided informed consent, and all the methods were performed in accordance with the approved guidelines. In total, 211 ESBL-producing *E*. *coli* isolates were analyzed from 2014–2015.

### Identification and Antimicrobial Susceptibility Screening

The purified isolates were freshly cultured on blood agar plates at 37 °C for 18–20 hrs using a biosafety level-2 cabinet. The identity of the purified isolates was determined by MALDI-TOF (MALDI Biotyper Bruker Daltonics, USA) according to the manufacturer’s instructions as described in our previous study^37^. The calibration was performed using *E*. *coli* ATCC 8739 as a standard to validate the run. All the isolates were run in duplicate, and identity score of ≥1.9 with database spectra was considered as a correct identification. All the isolates were initially tested for antimicrobial susceptibility using an automated VITEK-2 (BioMérieux, France) system with a specific AST-N291 card for ESBL-producing Gram-negative bacteria. The results were interpreted according to the guidelines of the CLSI^38^. The criteria of Magiorakos *et al*. was used to defined MDR, XDR and PDR isolates^39^.

### ERIC PCR, MLST and Whole Genome Sequencing

The study isolates were typed by Enterobacterial repetitive intergenic consensus (ERIC) PCR to identify genetic relatedness and to limit the number of strains for further investigations. ERIC PCR was performed in 25 µl volumes containing 1 µl (10 µM) of each primer (Table 1), 12.5 µl Go Taq® Green Master Mix (Promega, USA), 1.5 µl DNA template and 9 µl nuclease free water (Promega, USA) following the PCR conditions described by Versalovic *et al*.^40^. As previously described, an Achtman MLST scheme based on the internal fragment amplification of housekeeping genes was used to type the ESBL-producing *E*. *coli* isolates with slight modification of the annealing temperature^41^. The allelic profiles and sequence types (STs) were retrieved from the BLAST search of amplified genes in the EnteroBase database (https://enterobase.warwick.ac.uk/species/index/ecoli).

Whole genome sequence was performed for the isolates were STs were not defined from the Achtman scheme. Genomic DNA of the isolates was sequenced on a MiSeq platform (Illumina, USA) with the paired-end strategy using a Nextera XT library kit (Illumina, USA) as described previously^42^. The ID and allelic profile of the novel ST was obtained from the EnteroBase after uploading the raw read files connected with the metadata.

### PCR of β-lactamase Genes

PCR and sequence analyses were performed to determine the genes responsible for the ESBL-phenotype in the ESBL-producers. PCRs for the *bla*_TEM_, *bla*_SHV_, *bla*_CTX-M_ and *bla*_OXA_ genes were conducted using previously described multiplex PCR primers and conditions^43^. The full-length *bla*_CTX-M_ gene was amplified from the ESBL-producing isolates using the primers and conditions described by Pfeifer *et al*.^44^ and was sequenced with an ABI prism sequencer 3730 (Applied Biosystems, USA). The major groups (*bla*_CTX-M-1_, *bla*_CTX-M-2_, *bla*_CTX-M-9_ and *bla*_CTX-M-8/25_) were amplified from the *bla*_CTX-M_-positive isolates using group-specific primers according to Xu *et al*.^45^.

### Molecular Modeling and Docking

Once confirmed as *bla*_CTX-M_ gene sequences by BLAST, the different *bla*_CTX-M_ nucleotide sequences were translated to corresponding amino acid sequences using the ‘EXPASY translate tool’. Subsequently, the CTX-M amino acid sequences were subjected to multiple sequence alignments using CLUSTALW to detect any possible mutations, particularly in the active site Ω-loop, which is known to affect the substrate profile of CTX-M enzymes. Modeling was performed using the Swiss Model Server. The modeled structure (prior to docking) was verified using multiple validation programs, including VADAR, GeNMR and PROSESS. PDB structures of drugs/inhibitors were retrieved from Drug bank. The ligands (drugs/inhibitors) were docked into the active crevice of the modeled CTX-M-15 enzyme using AUTODOCK4.2^46^ with the aid of the ‘CLICK-BY-CLICK’-protocol^47^. The ligands included beta-lactam antibiotics (cefepime, cefotaxime and ceftazidime) as well as inhibitors (avibactam, clavulanate, tazobactam and sulbactam). All of the ligand structures were energy-minimized employing an MMFF94 force field. “Gasteiger charges” were added to the ligand atoms. Rotatable bonds were defined and nonpolar hydrogens were added. “Kollman charges”, essential hydrogens, and other relevant solvation parameters were also added. AUTOGRID was used to construct a grid of 40 Å^3^ with a spacing of 0.375 Å to target the active crevice of the CTX-M-15 enzyme model. The values of x, y, and z grid coordinates used to target the CTX-M-15 binding pocket were −4.1755, −2.4075, and 8.8905, respectively. Docking experiments used the ‘Solis & Wets local search method’ and a Lamarckian genetic-algorithm (LGA) and incorporated 100 runs each. Each run was programmed to perform 2.5 million “energy-evaluations” prior to termination. Discovery Studio (BIOVIA, San Diego) was used for molecular visualization of the docked complexes and the preparation of figures. Docking energies (ΔG, kcal/mol) were calculated for each of the binding pairs. Accordingly, we narrowed the observations down to few very efficient drugs and CTX-M-inhibitors with reference to the Saudi setting.

### Phylogenetic and Statistical Analysis

BioNumerics (V 7.6.0) from Applied Maths was used to analyze the ERIC PCR fingerprinting results, and a dendrogram was constructed using unweighted pair group with arithmetic mean (UPGMA). The default parameters of a 1% tolerance and an 85% similarity index were considered for clustering the isolates based on fingerprinting data. Pearson chi-square or likelihood ratio were used with a significance threshold of p < 0.05.

## Electronic supplementary material

Supplementary Figures
