# Supplementary material for: Molecular characterization, antimicrobial resistance and clinico-bioinformatics approaches to address the problem of extended-spectrum β-lactamase-producing Escherichia coli in western Saudi Arabia
Source: Sci Rep. 2018 Oct 4;8:14847. doi: 10.1038/s41598-018-33093-8 (PMC6172265; doi:10.1038/s41598-018-33093-8)
Supplement: Supplementary file 1 — Supplementary Figures [file 41598_2018_33093_MOESM1_ESM.pdf]

Molecular characterization, antimicrobial resistance and clinico-bioinformatics approaches to address the problem of extended-spectrum β-lactamase-producing *Escherichia coli* in western Saudi Arabia

Muhammad Yasir[1*], Abeer M. Ajlan[1,2], Shazi Shakil[3,4,5], Asif A. Jiman-Fatani[6,7], Saad B. Almasaudi[2], Muhammad Farman[1,2], Zainah M. Baazeem[1], Rnda Baabdullah[1], Maha Alawi[6,8], Nabeela Al-Abdullah[8,9], Nashat A. Ismaeel[6], Hani A. Shukri[7], Esam I. Azhar[1,5]

[1]Special Infectious Agents Unit, King Fahd Medical Research Center, King Abdulaziz University, Jeddah, Saudi Arabia

[2]Biology Department, Faculty of Science, King Abdulaziz University, Jeddah 21589, Saudi Arabia

[3]Center of Innovation in Personalized Medicine, King Abdulaziz University, Jeddah, Saudi Arabia

[4]Center of Excellence in Genomic Medicine Research, King Abdulaziz University, Jeddah, Saudi Arabia

[5]Department of Medical Laboratory Technology, Faculty of Applied Medical Sciences, King Abdulaziz University, Jeddah, Saudi Arabia

[6]Department of Medical Microbiology and Parasitology, Faculty of Medicine, King Abdulaziz University, Jeddah, Saudi Arabia

[7]Clinical and molecular microbiology laboratories King Abdulaziz University Hospital, Jeddah, Saudi Arabia

[8]Infection Control & Environmental Health Unit, King Abdulaziz University Hospital, King Abdulaziz University, Jeddah, Saudi Arabia

[9]Department of Public Health, Faculty of Nursing, King Abdulaziz University, Jeddah, Saudi Arabia

[*]Corresponding author: Muhammad Yasir
E mail: yasirkhattak.mrl@gmail.com

Ph: 00966563202241

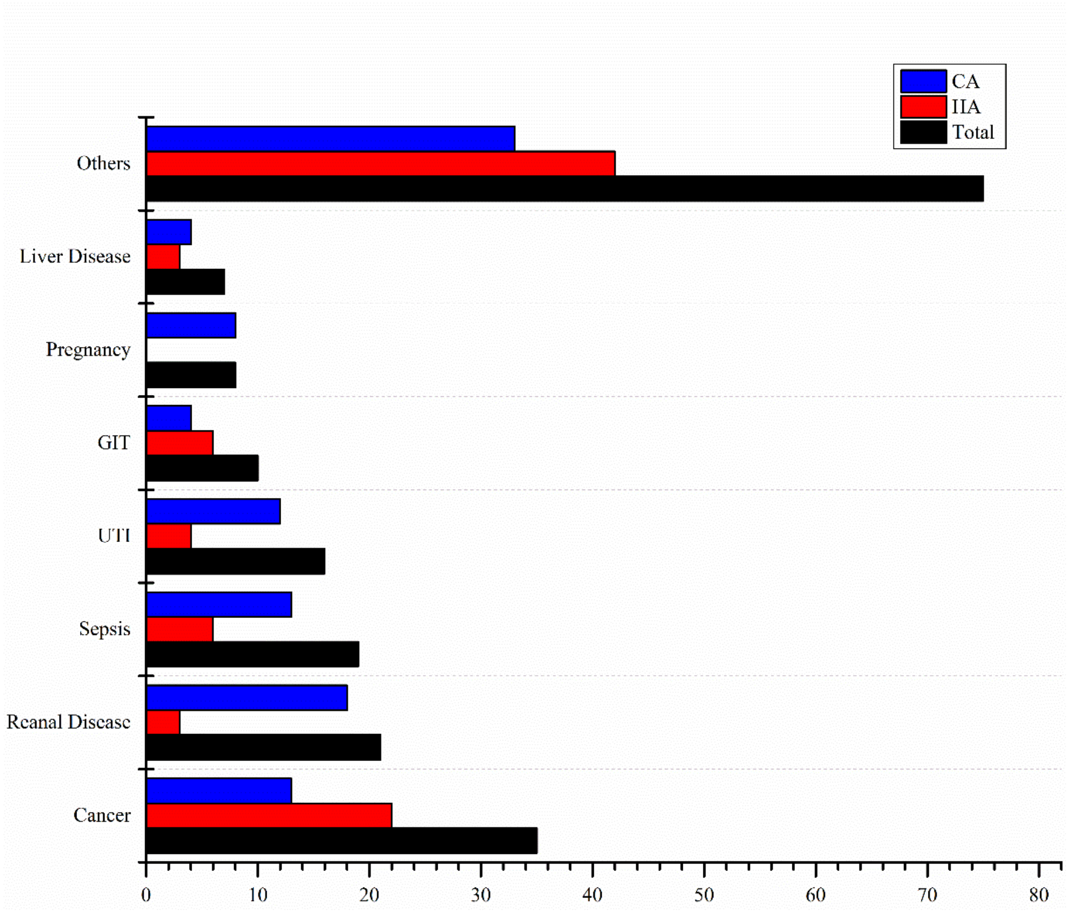

Fig. S1. Distribution of the community acquired (CA) and hospital acquired (HA) infection of ESBL-positive *E. coli* in different disease patients. The x-axis values expressed the number of isolates.

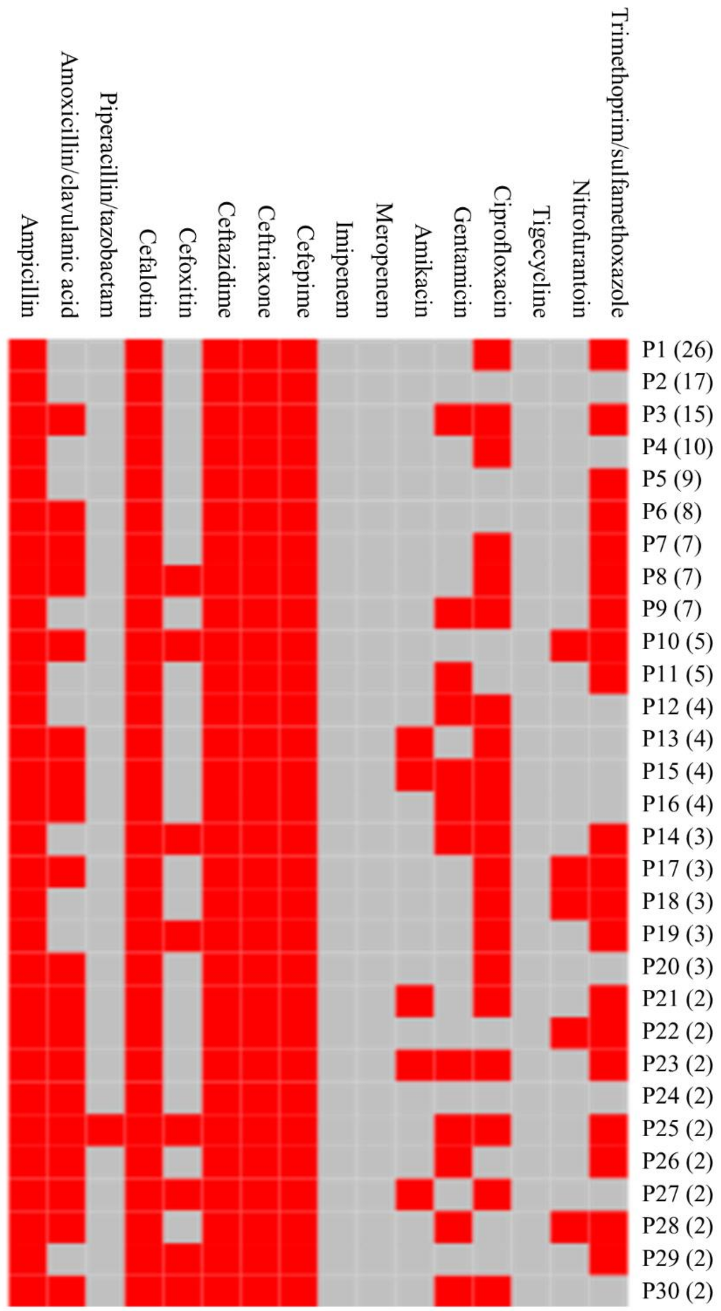

Fig. S2. Antimicrobial resistance and susceptibility patterns among the studied ESBL-positive *E. coli* isolates.

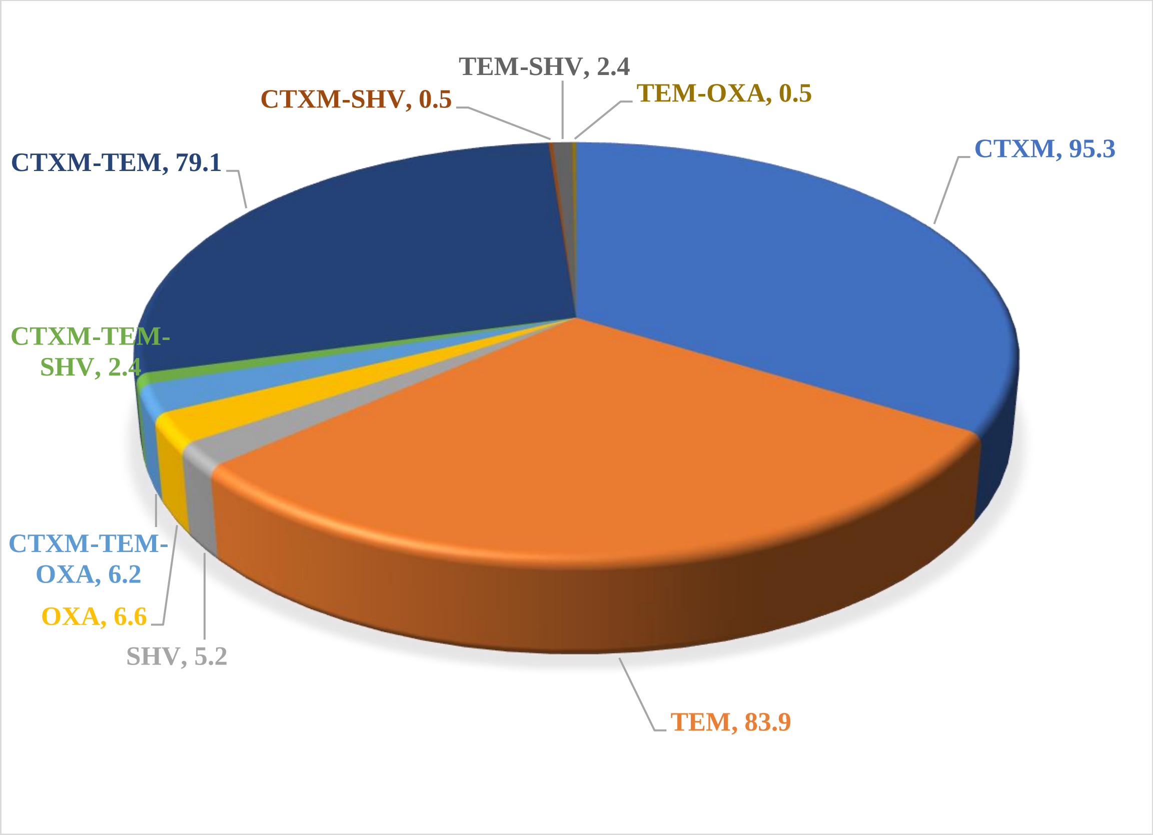

Fig. S3. Percentage distribution of the ESBL genes in the ESBL-positive *E. coli* isolates.

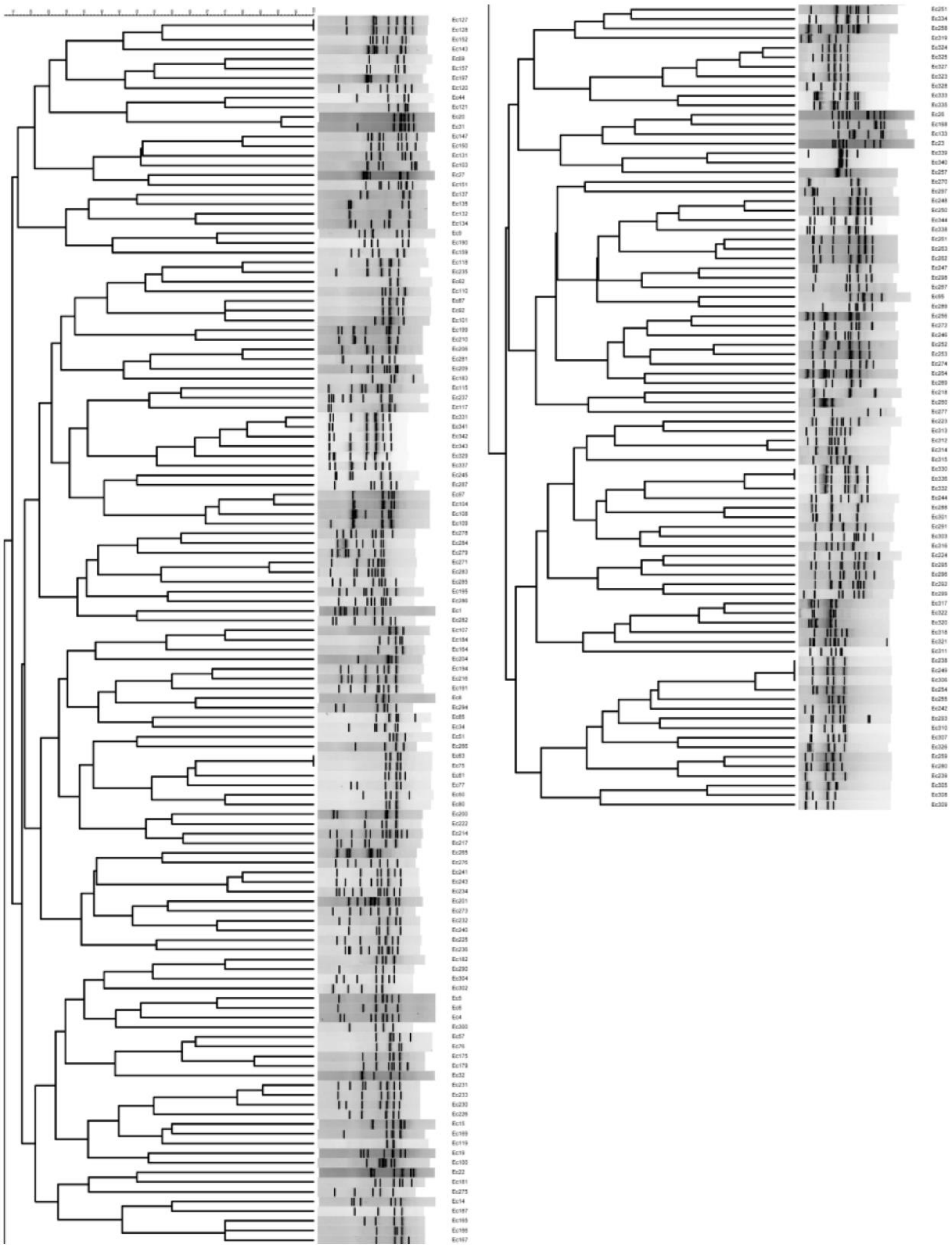

Fig. S4. UPGMA dendrogram of the ERIC-PCR bands pattern of the ESB-positive *E. coli* isolates.

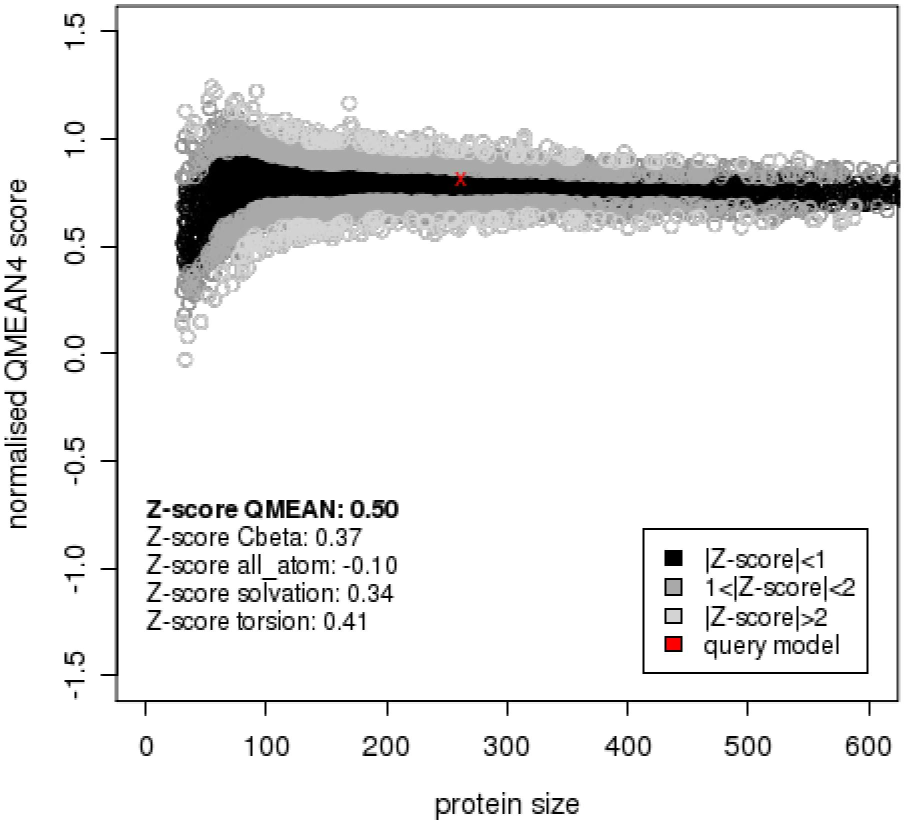

Fig. S5. Plot representing normalized "QMEAN4" scores versus protein-size to assess the bacterial enzyme model reliability with reference to a non-redundant set of PDB-structures.

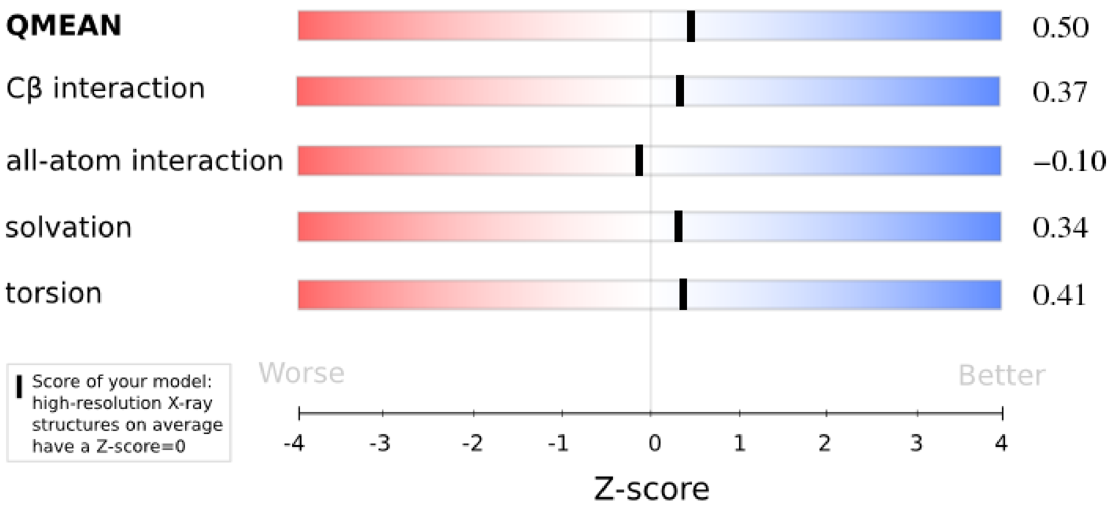

Fig. S6. ‘Slider Plot’ representing Z-score values for Cß interaction, all-atom interaction, solvation and torsion corresponding to the bacterial enzyme model.
